# A preliminary construction of the practical model of children’s humanistic care from the perspective of Maslow’s hierarchy of needs theory

**DOI:** 10.3389/fmed.2026.1804361

**Published:** 2026-08-24

**Authors:** Haini He, Jiao Chen, Yu Lu, Li Ma

**Affiliations:** 1 Department of Pediatrics, West China Second University Hospital-Tianfu·Sichuan Provincial Children’s Hospital, Meishan, Sichuan, China; 2 Department of Pediatrics, West China Second University Hospital, Sichuan University, Chengdu, Sichuan, China; 3 Department of Pediatrics, Key Laboratory of Birth Defects and Related Diseases of Women and Children of MOE, West China Second University Hospital, Sichuan University, Chengdu, Sichuan, China

**Keywords:** children, humanistic care, Maslow’s hierarchy of needs theory, model, semi-structured interviews

## Abstract

**Objective:**

This study aims to initially construct the model for children’s humanistic care practice based on Maslow’s hierarchy of needs theory.

**Methods:**

This study used purposive sampling, selected 16 nurses from the pediatric department of the children’s hospital who had previously cared for a 12-year-old child with congenital heart disease (*Lucas*). The study utilized the thematic analysis method and conducted the investigation through face-to-face semi-structured interviews, each lasting 20–30 min.

**Results:**

Through initial coding and theme analysis, this study identified 11 sub-themes: background assessment, active responding, attentive companionship, emotional soothing, provision of support, maintaining comfort, providing feedback, respectful listening, achievement motivation, knowledge acquisition and sustaining belief. Based on Maslow’s hierarchy of needs theory, these sub-themes were classified into 4 main categories (safety needs, love and belonging needs, esteem needs and self-actualization), and a preliminary practical model of children’s humanistic care was developed.

**Discussion:**

This study preliminary formed a practical model of children’s humanistic care based on Maslow’s hierarchy of needs theory, aiming to address the physical and psychological needs of children.

**Conclusion:**

Based on Maslow’s hierarchy of needs theory, this study preliminary formed a practical model of children’s humanistic care, including establishing connection, providing assistance, encouraging expression, and promoting health.

## 1 Introduction

Humanistic care, as an important part of modern medical service, adheres to the people-oriented idea and sticks to the principle of “patient-centered,” reflecting respect for patients and awe of life (1–3). Research showed that the essence of “patient-centered” was “need-centered” (4). Therefore, Maslow’s hierarchy of needs theory could provide the classic framework for understanding patient needs. According to humanistic nursing theory, humanistic care was characterized by the interactions between healthcare workers and patients to promote quality of life (5). However, previous studies reported that lacking humanism might prevent effective communication between nurses and patients, might cause the relationship between staff and patients to deteriorate, might result in a loss of trust between healthcare workers and patients (1, 6). At present, most studies have focused on the caring abilities of nurses for adults, while some studies have reported on humanistic care education for nursing undergraduates (7, 8). This indicated that previous studies had paid less attention to humanistic care for children and lack qualitative research on case-based humanistic care for children.

Maslow’s hierarchy of needs theory had divided human needs into five levels, namely, physiology, safety, love and belonging, esteem and self-actualization (9). It advocated nursing care based on the patient of stage to maximally meet individual pathological and psychological needs. Moreover, unlike adult patients, children were in a stage of rapid physical and mental development. Their needs had distinct hierarchical and developmental characteristics (10, 11). Studies showed that the five levels of needs proposed by Maslow were all manifested to varying degrees in children, and their physiological needs, safety needs, and love and belonging needs showed a clear progressive trend as they grew older (12). This implied that the paths for meeting children’s needs were naturally in line with Maslow’s hierarchical assumptions. Therefore, Maslow’s hierarchy of needs theory could serve as the reference for the hierarchical needs in pediatric humanistic care, and provided specific operational basis for humanistic care.

However, it was acknowledged the Maslow’s hierarchy of needs theory had strict sequential limitations (13, 14). Different studies had indicated that patients might experience multiple needs simultaneously during hospitalization (15–17). Nevertheless, this study did attempt to use Maslow’s hierarchy of needs theory as the guideline without strictly adhering to its sequential order. Instead, this study provided specific humanistic care behaviors based on the different dominant needs of the children at different disease stages. Therefore, this study selected a successful and specific case of humanistic care as the basis for the interview. By introducing this specific case, the study collected the actual and expected humanistic care measures implemented by the nurses. The findings were then used to preliminarily construct a practical model of humanistic care for children.

## 2 Case report

This study selected a 12-year-old child with congenital heart disease (CHD) from a certain children’s hospital. To protect privacy, the pseudonym *Lucas* was used for the child throughout the entire text. *Lucas* underwent corrective surgery for double-outlet right ventricle with bioprosthetic pulmonary valve replacement in hospital due to congenital heart disease: double-outlet right ventricle (Tetralogy of Fallot Type). *Lucas* had recurrent fever for over 1 month after surgery. Considering the diagnosis of infective endocarditis, *Lucas* was successively treated with anti-infective treatments including meropenem combined with vancomycin, micafungin, and polymyxin B. However, despite this treatment, fever recurred persistently. Later, the antibiotics were adjusted to linezolid, imipenem-cilastatin and rifampicin for anti-infection. The body temperature returned to normal, and *Lucas* was transferred to our department for continued treatment. *Lucas* was conscious when admitted to the hospital. The median sternotomy incision on the anterior chest was well-healed with no signs of bleeding or exudate. Heart sounds were strong with regular rhythm, and grade I-II systolic murmur was audible at the parasternal left 2nd to 3rd intercostal spaces.

In 2022, *Lucas* underwent burr hole surgery to aspirate pus due to “brain abscess.” Following anti-infective treatment, intracranial pus aspiration was performed twice more. Since birth, *Lucas* has had an inguinal hernia with contents descending into the scrotum. The hernia has become more prominent with increased intra-abdominal pressure but has not become incarcerated to date. Current examinations: Electrocardiogram: (a) Sinus rhythm; (b) Right axis deviation (+141°); (c) Complete right bundle branch block. Echocardiography revealed the following findings: residual ventricular-level shunt, accelerated pulmonary artery forward flow, tricuspid regurgitation (mild to moderate), and mitral regurgitation (mild). After 28 days of anti-infection treatment and nursing care, *Lucas* recovered and was discharged from the hospital.

*Lucas* has become somewhat withdrawn due to parents’ divorce and was reluctant to communicate with others. Physically, *Lucas* presented with a complex, protracted illness involving multiple hospitalizations and recent cardiac surgery, resulting in physical pain. In terms of social support, *Lucas’* family had faced a heavy financial burden due to multiple surgeries since birth. *Lucas’s* parents were not consistently present to provide care, and grandmother was the primary caregiver. In the situations described above, *Lucas’s* positive outcomes from nursing perspective included: (a) infection indicators continuously decreased after admission, with no new infections occurring; (b) treatment compliance steadily improved; (c) progressed from reluctance to speak to engaging willingly with nurses; (d) Emotional state stabilized from initial instability.

## 3 Materials and methods

### 3.1 Subjects

This study used purposive sampling, selected 16 nurses from the pediatric department of the children’s hospital who had previously cared for *Lucas*. Face-to-face semi-structured interviews were conducted, with each interview lasting 20 ~ 30 min. The interview duration was based on clinical realities, as the interviews were conducted during duty hours to minimize disruption to patient care. Saturation was determined when two consecutive interviews yielded no new themes. Data saturation was preliminarily reached at the 14th interviewee. To ensure and confirm theoretical saturation, interviews with all 16 planned participants (coded S1 ~ S16) were completed. This study was conducted in accordance with the Declaration of Helsinki and was approved by the Ethics Committee of Sichuan Provincial Children’s Hospital.

### 3.2 Data collection

The interview guide was ultimately determined through internal discussion within the research team, and it specifically included the following questions: (a) Regarding *Lucas’s* hospitalization, what humanistic care behaviors did you implement for him? (b) What were the most challenging aspects during the implementation process? (c) Did you have any regrets concerning his care—things you wished to do for him but could not, and why? (d) Beyond the aforementioned humanistic care behaviors, what other actions have you taken in caring for other pediatric patients?

**Figure 1:**
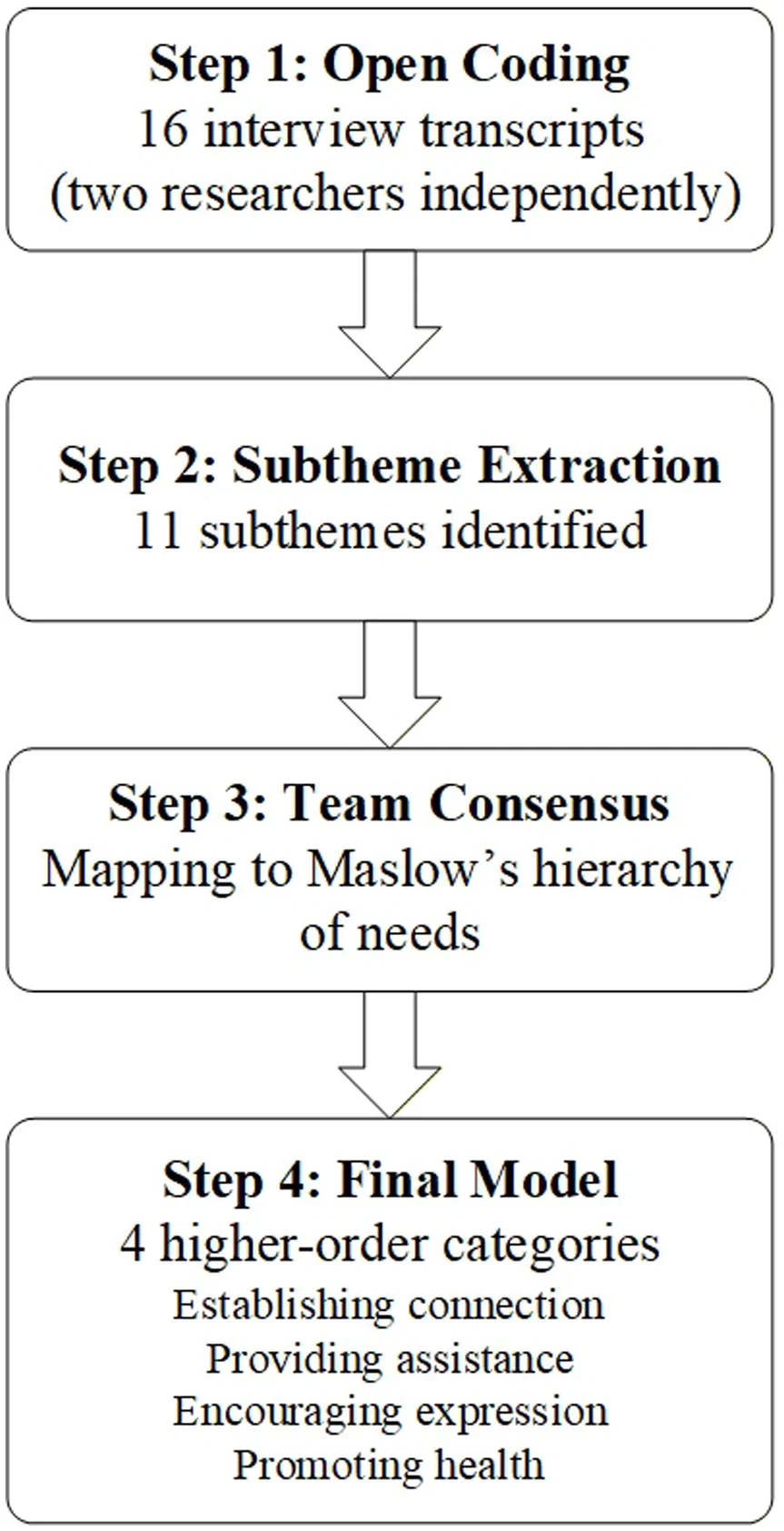
Thematic development diagram. The figure illustrates the four-step analytical process: Open coding of 16 interview transcripts (Step 1), identification of 11 sub-themes (Step 2), team consensus-based mapping to Maslow’s hierarchy of needs (Step 3), and the final model comprising four higher-order categories: Establishing connection, Providing assistance, Encouraging expression, and Promoting health (Step 4).

The thematic synthesis was conducted in four steps: (a) two researchers independently coded the interview transcripts using open coding; (b) similar codes were grouped into 11 subthemes; (c) through team discussion and consensus, these 11 subthemes were mapped to Maslow’s hierarchy of needs theory; (d) the final practice model was preliminary developed through iterative review by all authors, resulting in four higher-order categories (Figure 1).

### 3.3 Data analysis

This study employed the theory-driven thematic analysis method (18), with Maslow’s hierarchy of needs theory as the priori analytical framework. Two researchers independently conducted manual coding of interview transcripts based on the coding framework.

#### 3.3.1 Construction of the coding framework

Maslow’s hierarchy of needs theory had categorized into five levels from the lowest to the highest: physiological needs, safety needs, love and belonging needs, esteem needs, and self-actualization. Based on the characteristics of clinical nursing practice and relevant literature, the research team constructed a coding framework applicable to this study and clarified the operational definitions of each need level within the research context. The specific coding framework was shown in Table 1.

**Table 1:** Coding framework based on Maslow’s hierarchy of needs theory.

Theoretical level	Contextualized operational definitions
Physiological needs	The basic nursing behaviors performed by nurses to maintain the child’s vital signs and ensure physical comfort encompass oral nursing, nutritional support, as well as rest and sleep care.
Safety needs	Behaviors performed by nurses to reduce children’s fear of needle procedures and establish a sense of safety in nursing care, including pre-procedural explanation, environmental safety assurance, minimization of invasive procedures, and child life therapy.
Love and belonging needs	Behaviors performed by nurses to establish emotional connection with children and to express care and affection, including companionship, comfort, daily greetings, and physical touch.
Esteem needs	Behaviors through which nurses respect the child’s dignity and value the child’s wishes and choices, including providing opportunities for expression, offering choices, treating the child equally, listening attentively.
Self-actualization	Behaviors that encourage children to take part in their own care, use their abilities, and maintain autonomy, including supporting decisions, promoting self-management, and building confidence.

#### 3.3.2 The process of implementing the code

Three interview transcripts were selected for inter-coder agreement testing. The inter-coder agreement was 82.0%, calculated as simple percentage agreement. Discrepancies were resolved through discussion until consensus was reached. Based on Maslow’s hierarchy of needs theory, sub-themes were identified and organized. All interviews were conducted by a trained researcher who maintained reflexive notes throughout the data collection and analysis process to recognize and minimize potential personal biases. Additionally, member checking was performed by returning interview summaries to participants for verification, ensuring that the findings accurately reflected their experiences and perspectives.

## 4 Results

### 4.1 Results of thematic analysis

In this study, initial coding was first performed on the interview transcripts, and the original data were broken down and conceptualized, resulting in 79 initial codes. Subsequently, the researchers performed comparative and inductive analysis, merged the initial codes, and ultimately formed 11 sub-themes. The 11 sub-themes included: background assessment, active responding, attentive companionship, emotional soothing, provision of support, maintaining comfort, providing feedback, respectful listening, achievement motivation, knowledge acquisition and sustaining belief (Table 2). Finally, the 11 sub-themes were categorized according to Maslow’s hierarchy of needs theory, and 4 main categories were derived: safety needs (establishing connection), love and belonging needs (providing assistance), esteem needs (encouraging expression) and self-actualization (promoting health) (Table 3). Specifically, “background assessment” and “active responding” were grouped under safety needs; “attentive companionship,” “emotional soothing,” “provision of support” and “maintaining comfort” were grouped under love and belonging needs; “providing feedback” and “respectful listening” were grouped under esteem needs; and “achievement motivation,” “knowledge acquisition,” and “sustaining belief” were grouped under self-actualization needs.

**Table 2:** Examples of categorization from initial coding.

Subcategories	Code	Initial concepts (with Direct quotes)
Background assessment	Family environment	S7: The child was in a special situation: his parents were divorced, and he was raised by his grandmother. His father was not very involved, often absent from the ward.
	Medical history assessment	S1: This child has undergone multiple surgeries in the past, and according to my discussion with the doctor, he might require further cardiac surgery in the future.
	Personality characteristics	S10: He appeared cold and sharp in words, yet deep down he yearned for care. Just a little warmth made him remarkably cooperative. This guarded disposition and social withdrawal likely stemmed from his upbringing and early environment.
	Financial status	S5: His family faced considerable financial hardship. I often found myself thinking of ways to reduce expenses for him whenever possible. His situation was particularly poignant—having been hospitalized repeatedly since early childhood, with his grandmother having exhausted all her savings on his medical care.
Active responding	Proactive inquiry	S1: Since his family members were frequently absent and he himself was rather quiet, I often took the initiative to ask if he had eaten, concerned that he might go hungry.
	Active explanation	S13: Whenever he asked me questions, I always responded with warmth and enthusiasm. Each time he pressed the bedside call button, I made sure to reach his bedside promptly, concerned that he might need assistance with something.
Respectful listening	Preservation of autonomy	S10: He was actually a 12-year-old, quite grown-up child. In my work, I often communicated with him from a friend’s perspective. During IV therapy, I would discuss the timing with him and let him decide when to proceed.
	Encouraging expression	S2: He would sometimes feel a bit down. I then invite him to stay with me at the nurses’ station while I worked, gently guiding him to express his thoughts. Mostly, I took on the role of a listener.
Attentive companionship	Being present	S3: Since his family members were often not around, I would have him wear his mask properly and stay with me at the nurses’ station. He often felt scared when alone during the night shift, so he would keep us company while we were on duty.
Emotional soothing	Soothing distress	S6: When the child would become upset and cry, I would hold him for a while to soothe his emotions.
	Alleviating Fear	S12: When caring for other children, I think what I did most frequently was probably providing comfort. For instance, some children were terrified of injections, and I would patiently calm them to help them feel better.
Provision of support	Acting within competence	S11: Whenever he needed anything, I did my best to help within my means. He often walked in the corridor, and each time I noticed he wasn’t wearing a mask, I would give him one to put on. Since only boiled water was available in the ward, whenever he needed warm water, I would get it for him at the nurses’ station. Sometimes when his father wasn’t there, he would often skip lunch. I really wanted to buy him a meal, but I was also concerned about potential stomach issues from outside food. If he seemed bored, I would bring him some books and toys from the ward to help pass the time.
Maintaining comfort	Providing comfort measures	S9: His bedsheets often got soiled, and I would promptly change them for him. During his hospital stay, the ward was not very crowded. To ensure his comfort and prevent infection, no other patient was placed in the adjacent bed.
Providing feedback	Positive reinforcement	S4: When he did not feel like talking, I prepared paper and a pen for him, encouraging him to draw instead. He was actually quite kind-hearted. On Mid-Autumn Festival, he even gave us several moon cakes, which deeply touched us at the time.
	Corrective feedback	S8: We had a suggestion box at the nurses’ station. On one occasion, he complained that the food from our hospital cafeteria was really unpalatable, so I relayed this feedback to the cafeteria.
Achievement motivation	Fostering the sense of achievement	S7: The previous department organized a Child Life activity. Initially, he said he did not want to go, but after some persuasion, he ended up having a great time and created a beautiful piece of work. During his hospitalization, I gave him a “Stamp Collection Booklet.” For every treatment he bravely underwent, he would receive a stamp. While he used to require a long mental preparation for injections, he later became very cooperative. Sometimes, when he followed me around, I would let him help by carrying a few things or doing small tasks. He was actually very happy and felt a strong sense of accomplishment from it.
Knowledge acquisition	Health education	S1: During each medication administration or IV infusion, I would clearly explain the medication precautions to both him and his family. He often ate instant noodles, and every time I saw it, I would tell him that it lacked nutrition and was not conducive to his recovery. When his father or grandmother was present, I would frequently remind them to pay close attention to observing the surgical incision on his chest. Since he had a long hospitalization, we emphasized to him daily the importance of strictly preventing cross-infection and consistently wearing a mask.
	Patient education	S8: During the last hand hygiene campaign organized by the department, I also encouraged him to participate and learn about proper hand washing techniques.
Sustaining belief	Maintaining confidence	S14: I think the most difficult thing was figuring out how to instill in him a greater sense of hope for life. Since his surgery did not lead to a complete cure, I wished for him to face life positively and view his illness with optimism, but I wasn’t sure what approach would be most appropriate.

**Table 3:** The distribution of codes for each demand level.

Maslow’s hierarchy of needs theory	Subcategories
Safety needs	Background assessment
	Active responding
Love and belonging needs	Attentive companionship
	Emotional soothing
	Provision of support
	Maintaining comfort
Esteem needs	Providing feedback
	Respectful listening
Self-actualization	Achievement motivation
	Knowledge acquisition
	Sustaining belief

### 4.2 Sub-themes and the categorization into Maslow’s hierarchy of needs theory

The sub-themes classified under the category of safety needs (establishing connection) included: background assessment and active responding. *His family faced considerable financial hardship. I often found myself thinking of ways to reduce expenses for him whenever possible. His situation was particularly poignant—having been hospitalized repeatedly since early childhood, with his grandmother having exhausted all her savings on his medical care(S5).* This indicates that nurses can proactively assess patients’ family and economic backgrounds and take personalized measures accordingly, such as cost-saving and proactive inquiries.

Love and belonging needs (providing assistance) included: attentive companionship, emotional soothing, provision of support and maintaining comfort. *Since his family members were often not around, I would have him wear his mask properly and stay with me at the nurses’ station. He often felt scared when alone during the night shift, so he would keep us company while we were on duty(S3). Whenever he needed anything, I did my best to help within my means. He often walked in the corridor, and each time I noticed he wasn’t wearing a mask, I would give him one to put on. Since only boiled water was available in the ward, whenever he needed warm water, I would get it for him at the nurses’ station. Sometimes when his father wasn’t there, he would often skip lunch. I really wanted to buy him a meal, but I was also concerned about potential stomach issues from outside food. If he seemed bored, I would bring him some books and toys from the ward to help pass the time(S11).* These findings reflect nurses’ efforts to respond to children’s need for belonging through emotional companionship, daily assistance, and environmental care.

Providing feedback and respectful listening emerged as the key sub-themes under esteem needs (encouraging expression). *He would sometimes feel a bit down. I then invite him to stay with me at the nurses’ station while I worked, gently guiding him to express his thoughts. Mostly, I took on the role of a listener(S2). When he did not feel like talking, I prepared paper and a pen for him, encouraging him to draw instead(S4). We had a suggestion box at the nurses’ station. On one occasion, he complained that the food from our hospital cafeteria was really unpalatable, so I relayed this feedback to the cafeteria(S8).* This reflects nurses’ respect for and responsiveness to the child.

Three sub-themes were identified for self-actualization (promoting health): achievement motivation, knowledge acquisition and sustaining belief *The previous department organized a Child Life activity. Initially, he said he did not want to go, but after some persuasion, he ended up having a great time and created a beautiful piece of work. During his hospitalization, I gave him a “Stamp Collection Booklet.” For every treatment he bravely underwent, he would receive a stamp. While he used to require a long mental preparation for injections, he later became very cooperative. Sometimes, when he followed me around, I would let him help by carrying a few things or doing small tasks. He was actually very happy and felt a strong sense of accomplishment from it(S7). During each medication administration or IV infusion, I would clearly explain the medication precautions to both him and his family. He often ate instant noodles, and every time I saw it, I would tell him that it lacked nutrition and was not conducive to his recovery. When his father or grandmother was present, I would frequently remind them to pay close attention to observing the surgical incision on his chest. Since he had a long hospitalization, we emphasized to him daily the importance of strictly preventing cross-infection and consistently wearing a mask(S1). I think the most difficult thing was figuring out how to instill in him a greater sense of hope for life. Since his surgery did not lead to a complete cure, I wished for him to face life positively and view his illness with optimism, but I wasn’t sure what approach would be most appropriate(S14).* These findings reflect nurses’ comprehensive support for children’s autonomy, health knowledge, and hope for life.

### 4.3 The practical model of children’s humanistic care

**Figure 2:**
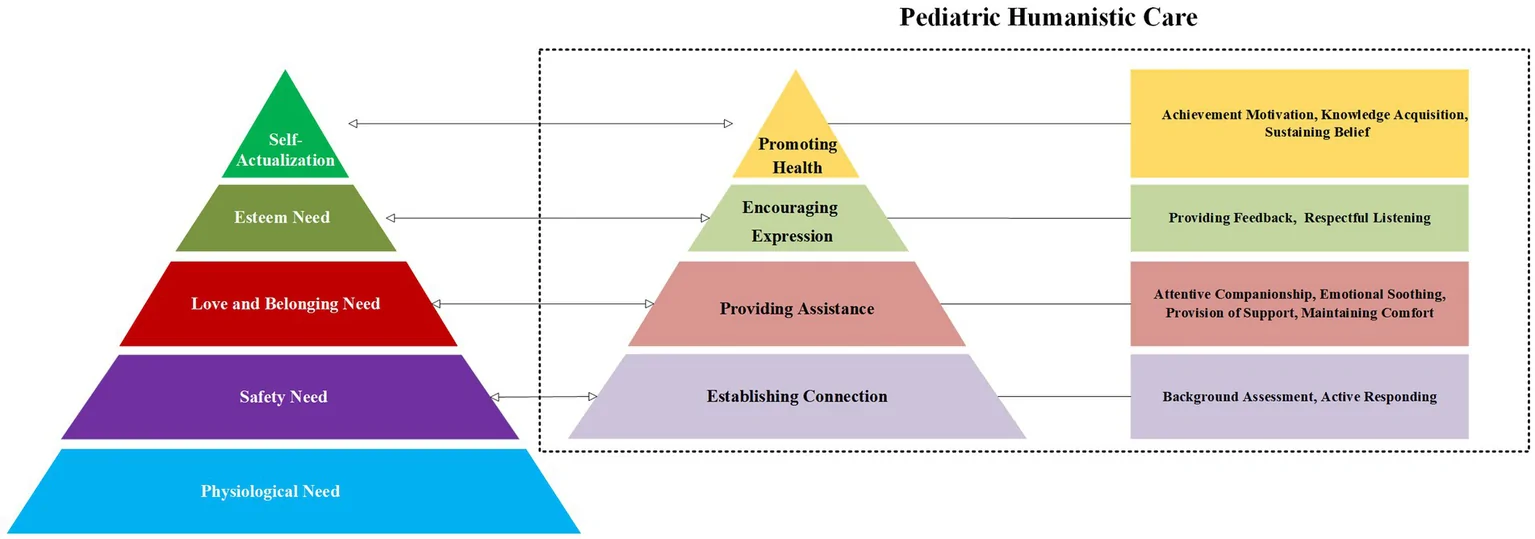
Children’s humanistic care practice model based on Maslow’s hierarchy of needs theory. The model comprises four higher-order categories derived from interview data (establishing connection, providing assistance, encouraging expression, and promoting health), corresponding to Maslow’s hierarchy of needs theory.

Based on the above 11 sub-themes and their classification within Maslow’s hierarchy of needs theory, this study preliminarily constructed the practical model of pediatrics humanistic care. The preliminary practical model of pediatrics humanistic care comprised establishing connection, providing assistance, encouraging expression, and promoting health (Figure 2).

## 5 Discussion

Based on Maslow’s hierarchy of needs theory and clinical practice, this study preliminary proposed a humanistic care practice model comprising four core dimensions: establishing connections, providing assistance, encouraging expression, and promoting health. These four dimensions corresponded closely to Maslow’s hierarchy of needs theory, clearly outlining the pathway of humanistic care in pediatric nursing. The preliminary model did not simply apply Maslow’s hierarchy of needs theory. Instead, it reorganized the four needs into actionable nursing practices based on how nurses actually care for children. Each dimension of the humanistic care practice model was accompanied by corresponding nursing behavior guidelines, such as “background assessment, respectful listening, and active responding.” These indicators were not only operational but reflect the core nursing philosophies of “patient-centered care” and “holistic care.”

In nursing practice, “caring encounter” has been conceptualized as a mutual interaction between nurses and patients (19). Rutherford et al. indicated that trust, as an intangible asset in nursing, influences the ability of nurses to establish deep and meaningful connections with patients. Mutual trust is the necessary condition for the effective nursing relationship (20). Therefore, establishing a good connection was the essential prerequisite for implementing humanistic care. Only when nurses established a sincere and trusting connection with a child could humanistic care be effectively delivered and generate a positive impact. The implementation of the humanistic care practice model transcended mere verbal communication and required that nurses engage deeply with children through empathetic listening, sincere concern and genuine care, thereby enhancing the warmth and depth of caregiving and embodying the intrinsic value of humanistic care.

The “establishing connection” and “encouraging expression” components emphasized in the humanistic care practice model proposed in this study fundamentally relied on nurses’ capacity for empathy. Previous studies have reported that empathy included both cognitive and affective dimensions, involving the ability to understand and convey patients’ inner experiences and perspectives (21, 22). At the same time, nurses with empathy could also better manage their emotions and consider the needs of the children from multiple aspects (23). Consistent with the statement of S10 in this study, “He appeared cold and sharp in words, yet deep down he yearned for care. Just a little warmth made him remarkably cooperative.” Based on the humanistic care practice model, pediatric nurses could more accurately understand and assess the needs of children, thereby establishing effective therapeutic relationships and providing more personalized and targeted care (24–26). Humanistic care was a constantly evolving process (27). The humanistic care practice model of this study regarded compassion, dignity, and the principle of humanistic care as the core of medical care services, in order to establish continuous and effective communication between nurses and patients (28, 29). Therefore, the nurses needed to dynamically adjust the humanistic care strategies based on the changes in a child’s physiological condition, psychological response, and family support system.

Moreover, when providing humanistic care, nurses were required to implement differentiated care strategies based on the age and developmental characteristics of children. For infants and toddlers, nurses used gentle tones and non-verbal tactile techniques to establish the sense of security. For preschool children, nurses should use simple language appropriate to their cognitive level to transform unfamiliar concepts into safe and understandable stories. For school-aged children, nurses should provide clear, gradual and honest explanations, encourage questions and respond sincerely, using communication to alleviate anxiety. For adolescents, nurses should focus on their emotional and psychosocial needs during continuous care (30). In addition, nurses have provided humanistic care for hospitalized children, and the subjects have often exhibited “dual characteristics,” requiring the satisfaction of both the children’s and their parents’ emotional needs (31). The hospitalization of children had caused their parents to experience worry, illness-related uncertainty, and caregiving stress (32). Therefore, nurses need to establish the good connection with the parents of the children in order to obtain more accurate information about the children’s illnesses and thus take more appropriate nursing care measures (33).

The current study had several limitations. First, although Maslow’s hierarchy of needs theory provided a useful framework for organizing subthemes, its use as an *a priori* framework may have constrained data interpretation. Therefore, we did not strictly adhere to its sequential order; rather, we used it as a reference to organize subthemes, while remaining open to emergent themes during coding. Second, this model was constructed based on the care provided by nurses to a 12-year-old boy. Therefore, its practicality for the implementation of humanistic care for younger children, including infants, needed further verification. Third, the interviews lasted only 20–30 min, which may have limited the depth of data exploration. Although we employed participant checking, independent coding, and team consensus to enhance trustworthiness, other strategies such as peer review and triangulation could be considered in future studies. Finally, this study used purposive sampling and was based on a single clinical case at a single institution, which might affect its transferability. Future multi-center research involving diverse pediatric populations is needed to evaluate the broader applicability of the proposed model. Further studies could also explore the development of counseling practice platforms for parents and family-level factors such as sibling support to extend the humanistic care framework. In conclusion, this study constructed a preliminary model for pediatric humanistic care practice. This preliminary model might serve as a reference for nurses to implement humanistic care and improve the care experience for children and their families.

## Data Availability

The original contributions presented in the study are included in the article/supplementary material, further inquiries can be directed to the corresponding author.
